# The chronological age at which small airway function starts to decline in a healthy Chinese population: a cross-sectional study

**DOI:** 10.3389/falgy.2025.1614899

**Published:** 2025-07-10

**Authors:** Lili Hou, Yonglu Guo, Yurong Zhang, Zhenghua Zhu, Ningfang Lian, Ying Gong, Bairong Du, Yuanyuan Liu, Jichen Qu, Xian Zhu, Lei Zhu

**Affiliations:** ^1^ Department of Respiratory and Critical Care Medicine, Jiuquan Branch of Shanghai General Hospital, Gansu, China; ^2^ Department of Respiratory and Critical Care Medicine, Shanghai General Hospital, Shanghai Jiao Tong University School of Medicine, Shanghai, China; ^3^ Department of Otolaryngology, Jiuquan Branch of Shanghai General Hospital, Gansu, China; ^4^ Department of Pulmonary and Critical Care Medicine, The First Affiliated Hospital of Fujian Medical University, Institute of Respiratory Disease, Fujian Medical University, Fujian, China; ^5^ Department of Respiratory and Critical Care Medicine, Zhongshan Hospital, Fudan University, Shanghai, China; ^6^ Department of Respiratory and Critical Care Medicine, Dunhuang General Hospital, Dunhuang, China; ^7^ Department of Critical and Pulmonary Medicine, Huadong Hospital, Fudan University, Shanghai, China

**Keywords:** aging, small airway, small airway dysfunction, spirometry, the area under receiver operating characteristic curves

## Abstract

**Background:**

Small airway function naturally declines with aging. However, the precise age this decline begins in healthy Chinese individuals remains unclear. This study aimed to identify the chronological age at which small airway dysfunction (SAD) initiates and its predictors within a healthy Chinese population.

**Methods:**

We conducted a retrospective, cross-sectional, multicenter analysis of 462 adults undergoing routine health checkups. The participants underwent standard spirometry and completed questionnaires. The onset age of SAD was determined using the area under the receiver operating characteristic curves (AUCs). Univariate and multivariate analyses identified relationships between variables and SAD.

**Results:**

Healthy individuals aged 55 years or older demonstrated SAD. Age was an important independent predictor for SAD (odds ratio, 1.107; *P* < 0.001). The AUC of age for predicting SAD was 0.771 (95% CI: 0.730–0.808), which significantly increased to 0.790 (95% CI: 0.750–0.826; *P* = 0.0347) after adjusting for covariates (body mass index and sex).

**Conclusion:**

We identified SAD predictors in a healthy Chinese population with environmental exposure. An age of 55 years or older was the threshold for predicting SAD in these healthy participants.

## Introduction

China's rapidly aging population presents a significant healthcare challenge, particularly concerning chronic respiratory diseases, including asthma and chronic obstructive pulmonary disease (COPD). The pathophysiology of these lung diseases may interact with normal aging physiology, exacerbating functional decline and worsening disease manifestations in older patients. Age-related changes influence lung structure and function, impacting diagnosis and treatment across different life stages. For instance, collagen fiber network changes with aging can cause alveolar duct dilation and enlargement (1–4) and decreased elastic recoil pressure (4), reducing expiratory flow and lung volume. However, the chronological age at which age-related pulmonary function decline begins can vary (5). For instance, forced expiratory volume in 1 s (FEV_1_) and forced vital capacity (FVC) show an accelerated decline between the ages of 65 and 93 (6), while residual volume increases by approximately 50% between the ages of 20 and 70 (7). Information regarding the aging of distal airways is limited. Studies indicate small airways in Americans aged 65 years or above can begin closing during quiet expiration (2, 4), and abnormalities have been reported around age 40 using computed tomography (CT) scans with parametric response mapping (8, 9). Furthermore, a positive correlation between maximal mid-expiratory flow (MMEF) <65% predicted and age >65 years was observed in the Swiss PneumoLaus cohort (10). Despite these insights, the starting age of small airway function decline in the healthy Chinese population remains unclear.

Small airways, defined as having an inner diameter of <2 mm, represent a “quiet zone” in healthy adult lungs, contributing minimal resistance to airflow (11, 12). However, small airway dysfunction (SAD) is regarded as a precursor to both asthma and COPD. Asthma involves the small airways at an early stage; chronic inflammation thickens these airways (13), significantly increasing resistance and worsening clinical symptoms (14, 15). Similarly, many patients with severe emphysema can develop chronic bronchitis (CB), and small airway pathology is associated with poorer outcomes, including higher mortality and less improvement in lung function following lung volume reduction surgery (16). In patients with COPD who experience frequent exacerbations, SAD measures are closely linked to neutrophilic inflammation in the small airways (17).

Combining SAD with fractional exhaled nitric oxide can predict airway hyperresponsiveness (AHR) in patients with chronic cough (18) or mild asthma (19). This combination is also valuable for early detection, monitoring, and prediction of acute exacerbations in COPD and asthma–COPD overlap (20). Considering the impact of age on small airway function, stratification based on the physiological age of patients with SAD is necessary to reduce the rates of underdiagnosis and misdiagnosis of asthma (21, 22) and COPD (23). A previous study already noted age-group differences in SAD variables when predicting AHR for asthma diagnosis (22). Therefore, determining the physiological age at which small airway function begins to decline is essential.

Despite their importance, detecting early physiological abnormalities of small airways is challenging because of their relative inaccessibility and small size. Various methods assess small airway function, including spirometry, the forced oscillation technique, the nitrogen washout test, peripheral wedged catheters, PET, and MRI. Spirometry is the most widely applied method, utilizing parameters such as MMEF, forced expiratory flow (FEF) between 25% and 75% of pulmonary volume, and the FEV in 3 s (FEV_3_)/FVC ratio to assess SAD.

For this study, we selected FEF_25%−75%_, FEF_50%_, and FEF_75%_ as key indicators based on recommendations from previous studies (18, 19, 22) and guidelines (24). SAD was diagnosed when at least two of these three indicators were <80% of predicted values (25), a criterion more suitable for the Chinese population (25). While FEF_50%_ and FEF_25%−75%_ show a strong correlation (*r* = 0.94) (26), FEF_50%_ is considered more feasible and simpler as it does not require computation to identify small airway diseases (26). Therefore, FEF_50%_ is considered an acceptable spirometric parameter to distinguish SAD from normal small airway function.

Provided that FEF_75%_ is <80%, this study adopted an FEF_50%_ cutoff value lesser than 80% to define the presence of SAD (27). We aimed to determine the starting age of SAD in healthy Chinese adults with normal radiography or chest CT scans. This will provide crucial insights to formulate age-stratified diagnostic criteria for asthma (22) or COPD, ultimately benefiting efforts to reduce misdiagnosis and missed diagnoses in China.

## Materials and methods

### Study design and population

In this retrospective, cross-sectional, multicenter, observational study, we recruited 1,320 health checkup adults from January to December 2017, January to August 2018, and January to December 2024. All participants underwent health checkups at the medical examination department. From this pool, we enrolled 462 adults by extracting their electronic medical databases records from Zhongshan Hospital affiliated with Fudan University, the First Affiliated Hospital of Fujian Medical University, and the Jiuquan Branch of Shanghai General Hospital. Our sample size was calculated using the events per variable (EPV) criterion for logistic regression, with a target of 10–20 events per predictor to minimize overfitting. In total, 190 participants were recruited from Shanghai (65 with SAD), 168 from Fuzhou (58 with SAD), and 104 from Jiuquan (30 with SAD).

The inclusion criteria for this study were as follows: age, 18–80 years; ability to complete the questionnaire and spirometry tests; normal chest x-ray or CT results; normal routine blood and biochemical results; and no self-reported symptoms such as cough, sputum production, chest tightness, or shortness of breath in daily life. The exclusion criteria included the following: cigarette smoking; exposure to particles or engaging in occupations with a potential respiratory hazard; underweight (<18.5 kg/m^2^), overweight (25.0–30.0 kg/m^2^), or obese (>30.0 kg/m^2^); respiratory tract infections in the previous 8 weeks; a history of chronic cough, sputum, dyspnea on exertion, chest tightness, or wheezing; or comorbid severe systemic diseases, including asthma, COPD, and interstitial lung disease. All healthy participants underwent standard spirometry tests, chest radiography, chest CT, and follow-up via telephone interviews. Demographic data and pulmonary function parameters were extracted from EMD records and health checkup patient files. This study was approved by the Ethics Committee of the Institutional Review Board of Jiuquan Branch of Shanghai General Hospital (no. 2025JQ001).

### Spirometric measurements

Among methods for assessing small airway function, impulse oscillometry (IOS) is the gold standard for detecting SAD. IOS does not require forced expiration, which can affect small airway closure, instead providing a completely effort-independent evaluation of respiratory system mechanics and revealing abnormalities across different lung regions (20). Given the simplicity of oscillometric tests, they can be especially valuable for assessing lung function in older adults (4). Notably, in patients with COPD, IOS can detect SAD earlier, even before spirometry identifies abnormalities (28). Spirometry is limited in identifying SAD because of its heavy reliance on FVC (29). In patients with reduced FVC, FEF measurements can be significantly compromised, potentially underestimating small airway involvement (29). However, the disadvantages of IOS in measuring small airway function include the lack of unified standards, absence of predicted values, and inability to distinguish between obstructive and restrictive airway diseases. Additionally, owing to equipment and operational limitations, IOS tests have not been performed in many medical institutions. In fact, none of the patients in our three research centers underwent IOS testing during their physical examinations. Because of these practical challenges, we relied on spirometry parameters to assess the small airway function.

All technicians involved in this study received standardized, effective training and held national pulmonary function standardization training certificates. Well-trained technicians performed the spirometry tests between 8 a.m. and 11 a.m. using an MS-PFT spirometer (Jaeger, Germany, or SensorMedics, Yorba Linda, CA, USA), obtaining triplicate measurements. These tests were performed according to the standards and recommendations of the American Thoracic Society/European Respiratory Society (30, 31).

We reviewed and analyzed seven pulmonary function parameters (PFPs): FVC, FEV_1_, FEV_1_/FVC (FEV_1_%), PEF, FEF at 25% of FVC exhaled (FEF_25%_), FEF at 50% of FVC exhaled (FEF_50%_), and FEF at 75% of FVC exhaled (FEF_75%_). Most of these were presented as percentages of predicted values. The expected values for lung function parameters were based on the prediction equation for East China. An FEF_50%_ cutoff value of <80% predicted indicated the presence of SAD. FEV_1_/FVC values were presented as absolute values.

### Outcomes

Our primary outcome was to determine the threshold age for predicting SAD in a healthy Chinese population. The secondary outcome involved confirming the correlation between SAD and age, body mass index (BMI), FEF_50%_, and sex.

### Statistical analysis

Normality of the data distribution was determined using the Kolmogorov–Smirnov test. Normally distributed data are presented as mean ± standard deviation, while non-normally distributed data are expressed as medians and interquartile ranges. For comparisons, we used the independent Student’s *t*-test (two-tailed) or the Mann–Whitney *U* test for independent samples. Count data were presented as percentages, with between-group comparisons performed using the chi-square test (*χ*^2^). Finally, we analyzed between-parameter correlations using Spearman's analysis. For our analysis of SAD, we binarized the FEF_50%_ variable, provided that FEF_75%_ was <80%: FEF_50%_ ≥ 80% was defined as “0” and FEF_50%_ < 80% as “1.” Similarly, sex was binarized, with male as “0” and female as “1.”

We then conducted a systematic variable screening. First, we performed variance inflation factor (VIF) analysis on all initially considered candidate variables, excluding those exhibiting multicollinearity (VIF ≥ 5). Subsequently, correlation validation was conducted for the retained indicators, which confirmed good independence among all indicators (all pairwise |*r*| < 0.7). Finally, these selected variables were incorporated into multivariate logistic analysis to identify independent factors. We then conducted a statistical analysis to assess the predictive value of these key indicators, both individually and in combination, for SAD in a Chinese population. From a professional and scientific standpoint, we ultimately selected independent variables that met both professional requirements and statistical significance.

Recognizing the potential confounding effects of age and sex, we adjusted for these covariates to enhance the accuracy of our SAD prediction. A receiver operating characteristic (ROC) curve was constructed to determine the thresholds of age, BMI, and sex for predicting SAD (FEF_50%_ < 80%), and we calculated their areas under the curve (AUCs). The optimal threshold was determined using Youden's index. All predictive models for SAD were constructed and validated using repeated fivefold cross-validation (five repeats). The average AUC of the five different cross-validation models and whole-model AUC using the entire dataset were calculated. The threshold for statistical significance for all analyses was set at *P* < 0.05. We performed analyses using SPSS software (version 22.0; IBM Corp., Armonk, NY, USA). MedCalc 19.0.4 was used for ROC construction and AUC comparison. Python was used for fivefold cross-validation. AUCs were compared using the *χ*^2^ test, following the method by Hanley and McNeil. Statistical significance was set at *P* < 0.05.

## Results

### Baseline characteristics in health checkup adults

**Figure 1:**
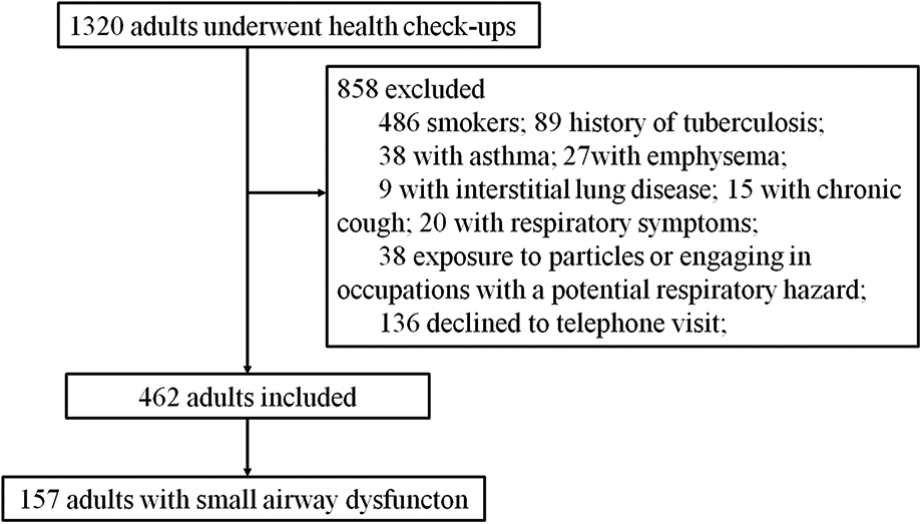
Flowchart of participant selection. This flowchart illustrates the selection process of participants from the adults who underwent health checkups.

A total of 1,320 health checkup patients were enrolled in the study, with 462 participants ultimately included after applying our inclusion and exclusion criteria (Figure 1). Among these, 157 (33.98%) exhibited SAD (FEF_50%_ < 80%), while 305 had normal small airway function. Of the 462 participants, 291 were aged 55 years or older, and 171 were younger than 55 years. Age-stratified sample distributions by decade and SAD incidence rates are shown in Table 1. SAD incidence progressively increased with age. Starting from 18 years and categorized every 10 years, the incidence rates of SAD were 0%, 0%, 6.38%, 31.51%, 46.77%, 62.12%, and 60% (Table 1). Notably, no statistically significant difference was observed in the distribution of SAD vs. normal small airway function within the age groups of 49–58 years and 79 years and above (Table 1). Table 2 details the baseline demographic characteristics and clinical features of the participants. Both FEF_25%_ and FEF_75%_ were significantly lower in participants with SAD than in those without it (*P* < 0.001). While FVC, FEV_1_, FEV_1_/FVC, and PEF were also significantly reduced in participants with SAD, most of these values remained within the normal range (all *P* < 0.01).

**Table 1:** The age-stratified sample distribution by decade and SAD incidence rates [*n* (%)], (defined by FEF_50%_ < 80%pred value).

Age (years old)	FEF_50_, %pred ≥ 80% (*n* = 305)	FEF_50_, %pred < 80% (*n* = 157)	*P*-value
18–28	25 (100)	0 (0)	**<0**.**001**
29–38	60 (100)	0 (0)	**<0**.**001**
39–48	44 (93.62)	3 (6.38)	**<0**.**001**
49–58	50 (68.49)	23 (31.51)	0.687
59–68	99 (53.23)	87 (46.77)	**<0**.**001**
69–78	25 (37.87)	41 (62.12)	**<0**.**001**
≥79	2 (40)	3 (60)	0.343

Bold font indicates statistical significance.

FEF_50%_, forced expiratory flow at 50% of the forced vital capacity.

**Table 2:** Features of health checkup adults by the presence of SAD (defined by FEF_50%_ < 80%pred value).

Characteristic variables	FEF_50_, %pred ≥ 80% (*n* = 305)	FEF_50_, %pred < 80% (*n* = 157)	*P*-value
Male (*n*/%)	176 (57.7%)	103 (65.6%)	0.100
Age, years	54.0 (35.0, 63.0)	64.0 (60.0, 70.0)	**<0**.**001**
BMI, kg/m^2^	23.62 ± 3.08	23.26 ± 2.81	0.219
Occupational composition (*n*,%)			
Teacher	75 (24.6)	42 (26.8)	0.613
Government staff	63 (20.7)	31 (19.7)	0.818
Doctor	56 (18.4)	29 (18.5)	0.977
High-altitude traveler	14 (4.6)	5 (3.2)	0.471
Policeman or policewoman	38 (12.5)	22 (14.0)	0.638
Office worker	59 (19.3)	28 (17.8)	0.694
Comorbidities (*n*, %)			
Hypertension	1 (0.33)	2 (1.27)	1.000
Paroxysmal atrial fibrillation	1 (0.33)	1 (0.64)	1.000
Thyroid nodule or cyst	12 (3.2)	5 (3.7)	0.685
Coronary artery disease	9 (3.0)	6 (3.8)	0.621
Pulmonary function variables			
FEF_25%_, % predicted	105.26 ± 16.42	82.52 ± 13.44	**<0**.**001**
FEF_75%_, % predicted	100.35 ± 28.61	66.53 ± 22.11	**<0**.**001**
FEF_25%−75%_,% predicted	95.40 (83.05, 107.75)	65.60 (55.26, 72.85)	**<0**.**001**
FVC, % predicted	100.22 ± 11.00	97.01 ± 11.58	**0**.**004**
FEV_1_, %predicted	105.69 ± 10.82	94.10 ± 8.49	**<0**.**001**
FEV_1_/FVC, %	85.50 ± 4.88	76.54 ± 3.37	**<0**.**001**
PEF, %predicted	98.70 ± 14.49	89.41 ± 12.34	**<0**.**001**
Blood parameters (reference value)			
RBCs (3.68–5.13 × 10^12^/L)	4.72 ± 0.76	4.72 ± 0.51	0.964
Hematocrit (33.5%–45.0%)	42.50 (39.75, 45.70)	42.70 (39.70,45.55)	0.899
Hemoglobin (113–151 g/L)	142.0 (133.0, 153.0)	141.0 (131.0,151.5)	0.441
Platelets (85–303 × 10^9^/L)	234.0 (196.5, 279.0)	241.0 (201.0, 282.0)	0.542
WBCs (4.0–10.0 × 10^9^/L)	6.46 (5.50, 7.82)	6.84 (5.63, 8.31)	0.158
Neutrophils (2.0–7.0 × 10^9^/L)	4.05 (3.19, 5.05)	4.07 (3.08, 5.04)	0.796
Lymphocytes(0.8–4.0 × 10^9^/L)	1.93 (1.57, 2.33)	2.10 (1.66, 2.56)	0.088
Eosinophils (0.02–0.5 × 10^9^/L)	0.10 (0.05, 0.18)	0.12 (0.06, 0.17)	0.061
Basophiles (0.00–1.00 × 109/L)	0.02 (0.01, 0.03)	0.02 (0.01, 0.03)	0.699
Monocytes (0.12–1 × 10^9^/L)	0.40 (0.31,0.5)	0.42 (0.33,0.52)	0.105
Neutrophils (40–70%)	60.64 ± 8.63	59.82 ± 8.83	0.337
Lymphocytes (20–40%)	30.51 ± 7.40	30.95 ± 8.22	0.555
Monocytes (3–10%)	6.00 (4.90, 7.10)	6.20 (5.00, 7.65)	0.319
Eosinophils (0.5–5%)	0.40 (0.70, 2.50)	1.60 (0.95, 2.60)	0.116
Basophiles (0–1%)	0.30 (0.20, 0.40)	0.40(0.30, 0.60)	0.317

Bold font indicates statistical significance.

BMI, body mass index; FEF_25%_, forced expiratory flow at 25% of forced vital capacity; FEF_50%_, forced expiratory flow at 50% of forced vital capacity; FEF_75%_, forced expiratory flow at 75% of forced vital capacity; FVC, forced vital capacity; FEV_1_, forced expiratory volume in 1 s; PEF, peak expiratory flow; WBCs, white blood cell; RBCs, red blood cell.

### Multicollinearity diagnosis among variables

After multicollinearity diagnosis and correlation validation, PEF, FEF_75%_, and age were identified as independent factors. However, for scientific and professional reasons, we only included age as an independent variable in the manuscript, based on the statistical results. None of the data are shown.

### Correlation of FEF_50%_ with age, BMI, and sex

**Figure 2:**
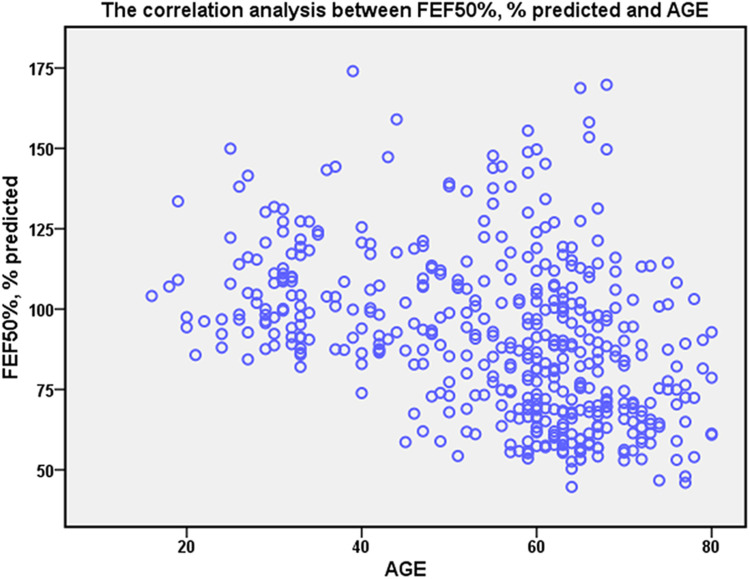
The correlation of FEF_50%_ with age. Spearman rank correlation analysis was applied to explore the correlation. The age has weakly correlation with FEF_50%_ (*r* = −0.439, *P* < 0.001).

Spearman analysis revealed a weak correlation of age with FEF_50%_ (*r* = −0.439, *P* < 0.001) (Figure 2). No significant correlation exists between FEF_50%_ and BMI (*r* = 0.093, *P* = 0.05) or sex (*r* = 0.039, *P* = 0.407).

### Threshold age and predictors for SAD in healthy adults

**Figure 3:**
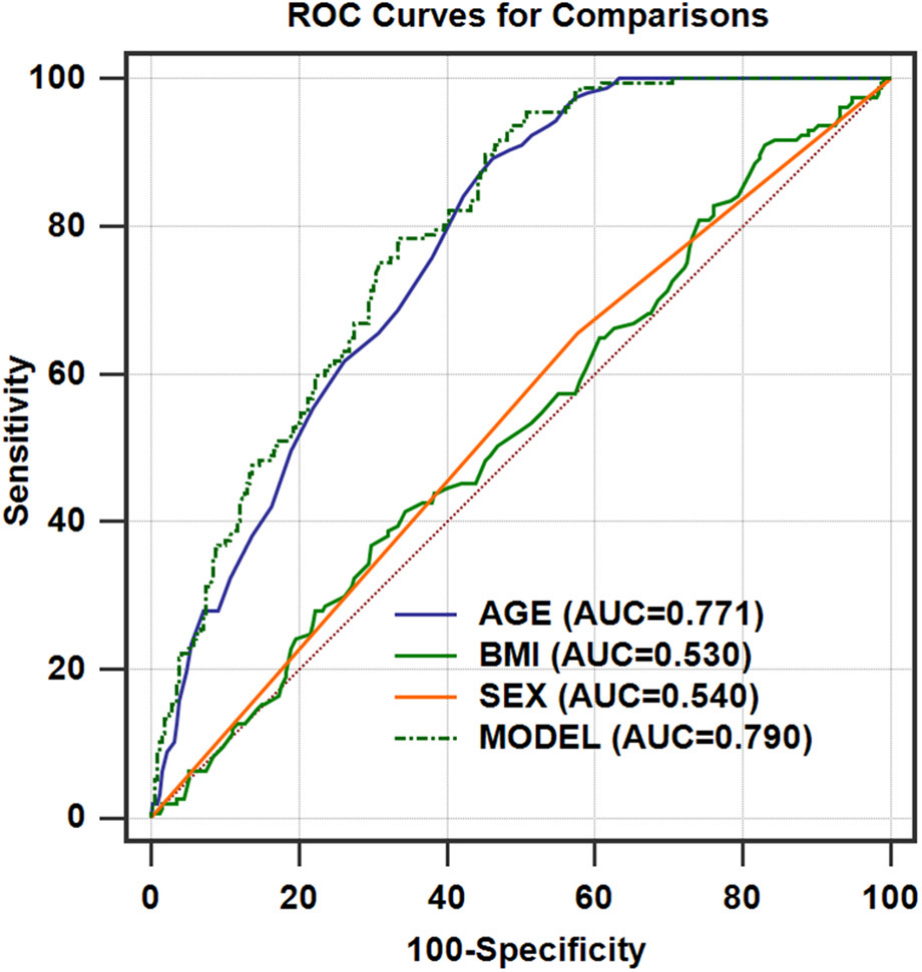
ROC curves for the models of age after adjusting for BMI and sex for predicting SAD in a healthy Chinese population (*n* = 462). AUC_model_ = 0.790 (95% CI, 0.750–0.826; *P* = 0.0347, compared with AUC_age_); AUC_age_ = 0.771 (95% CI, 0.730–0.808); AUC_BMI_ = 0.530 (95% CI, 0.483–0.576); AUC_sex_ = 0.540 (95% CI, 0.493–0.586). ROC, receiver operating characteristic; AUC, area under the curve.

We identified **55 years** as the threshold age for screening SAD in healthy populations (Table 2). The AUC for age alone in predicting SAD was 0.771 (95% CI: 0.730–0.808). To adjust for the covariates (BMI and sex), we analyzed the ROC curve by combining age with sex and BMI (model) (Figure 3 and Table 3). We found that the AUC of the model increased to 0.790 (95% CI: 0.750–0.826), which was significantly higher than that of age alone (*P* = 0.0347).

**Table 3:** The age threshold for decline of small airway dysfunction (SAD) and its value in distinguishing SAD in healthy Chinese adults before and after adjusting for covariates (BMI and sex).

Characteristic variables (*n* = 462)	Cutoff values	AUC	Sensitivity %	Specificity %	PPV %	NPV %	Accuracy %	+LR	−LR
Age, years	55	0.771	89.2	53.8	49.8	90.6	65.8	1.93	0.20
BMI, kg/m^2^	26.5	0.530	91.1	17.1	36.1	78.8	42.2	1.10	0.52
Sex	–	0.540	65.6	42.3	36.9	70.5	50.2	1.14	0.81
Model	**–**	0.790	93.0	51.5	49.7	93.5	65.6	1.93	0.14

Cutoff values were selected using Youden’s index.

AUC, area under the curve; PPV, positive predictive value; NPV, negative predictive value; +LR, positive likelihood ratio; LR, negative likelihood ratio.

Furthermore, univariate and multivariate analyses revealed age as a strong independent factor for SAD in our healthy adult cohort (OR, 9.959; 95% CI: 5.703–17.389; *P* < 0.001) (Table 4).

**Table 4:** The contribution of independent variables and covariates to the screening of SAD in a healthy checkup adult cohort using univariate and multivariate analysis.

Univariable analysis				Multivariable analysis		
Variables	OR	95% CI	*P*-value	OR	95% CI	*P*-value
Age >55 years	1.102	1.077–1.128	**<0.001**	1.107	1.082–1.133	**<0.001**
BMI ≤ 26.5	0.960	0.899–1.025	0.219	0.94	0.851–1.000	0.230
Sex: female	–	–	–	–	–	–

Bold font was selected for multivariate analysis in the process of univariable analysis (*P* < 0.1).

Bold font indicates significant differences in the multivariate analysis (*P* < 0.05).

BMI, body mass index.

### Internal cross-validation of the final models

The error rates, comparing the average AUC and threshold from the five cross-validation models with the AUC and threshold of the complete dataset, were <0.05, indicating that the data model had a reliable predictive capacity across various datasets (Table 5).

**Table 5:** AUC, sensitivity, specificity, PPV, NPV, +LR, −LR, and error rates in 80/20 split-sample cross-validation of prediction models.

Characteristic variables	Age												
	Training1	Test 1	Training2	Test 2	Training3	Test3	Training4	Test4	Training5	Test5	Average	Whole model	Error rate
AUC	0.772	0.765	0.761	0.810	0.779	0.736	0.776	0.752	0.766	0.784	0.769	0.771	0.26%
Sensitivity (%)	0.864	0.938	0.896	0.688	0.897	0.581	0.921	1	0.881	0.936	0.828	0.892	7.18%
Specificity (%)	0.545	0.591	0.541	0.787	0.570	0.771	0.521	0.459	0.521	0.607	0.643	0.538	19.52%
PPV	0.493	0.546	0.500	0.629	0.518	0.563	0.498	0.484	0.487	0.547	0.554	0.498	11.24%
NPV	0.8867	0.9474	0.9103	0.8276	0.9145	0.7833	0.927	1	0.8944	0.9487	0.901	0.9061	0.51%
+LR	1.899	2.288	1.952	3.226	2.084	2.530	1.920	1.849	1.837	2.378	2.454	1.9289	27.22%
−LR	0.2495	0.1059	0.1922	0.3971	0.1811	0.5443	0.1525	0	0.2287	0.1064	0.2307	0.2014	14.58%
Age (years)	57	56	56	64	56	64	55	49	56	57	58	56	3.57%

## Discussion

The main findings of this study are that older age is a crucial independent risk factor for SAD in healthy individuals, with those over 55 years being more susceptible.

This study showed that small airway function declined with age in healthy, non-smoking permanent residents. This contrasts with a Chinese national cross-sectional study which reported the highest prevalence of small airway obstruction (97.5%) among individuals aged 40–49 years, followed by a decrease in older age groups (32). Nevertheless, they observed that the likelihood of small airway obstruction increased with age, likely because they compared their data with a younger reference group. In that study, the prevalence of SAD in every 10-year age group among individuals aged under 70 years was significantly higher than what we reported (32). This might be attributed to the different diagnostic criteria for SAD used in our study and our efforts to balance the range of factors that influence small airway function.

Numerous factors can influence small airway function. Age, degree of urbanization, low educational attainment, active and passive smoking, use of biomass fuels, air pollution exposure, a childhood history of chronic cough, a childhood history of pneumonia or bronchitis, and a family history of respiratory diseases have all shown positively correlations with SAD (10, 29, 32). Both low and high BMI have also been linked to SAD (29, 32). While being female has sometimes been linked to a lower chance of developing SAD (29), Xiao et al. (32) revealed a significant correlation between female sex and a higher likelihood of developing SAD. Given these conflicting findings, establishing a true association between SAD and sex is challenging. Genetic polymorphisms are associated with an early decline in lung function following normal growth (33). Individuals with a higher percentage of African ancestry exhibit lower lung function than those with a lower percentage (34). This racial susceptibility in SAD helps explain why White individuals are more prone to emphysema and CB than Black individuals. However, a recent study suggests that removing race-based reference values could decrease the underestimation of disease severity in African Americans with COPD (35). Previous findings have reinforced the evidence that occupation, independent of smoking, is a risk factor for lung function decline (36). Our study excluded individuals with hazardous occupational exposures known to affect pulmonary function, thereby mitigating the impact of these factors on SAD to some extent. Despite our efforts to balance various confounding factors, we cannot entirely rule out the possibility that residual confounding factors may have influenced our findings.

Additionally, the physiological age threshold for detecting a decline in small airway function may vary depending on the measurement method used. Pathological and radiological changes in the small airways typically precede the changes detected by spirometry. Using parametric response mapping analysis of paired inspiratory/expiratory CT scans, normal aging contributes to SAD in non-smokers or ever-smokers in different subgroups, including individuals with or without airflow obstruction, regardless of the presence of respiratory symptoms (4, 8, 29, 37). Moreover, SAD, caused by aging, increases by 2.7% per decade, ranging from 3.6% (ages 40–50 years) to 12.7% (ages 70–80 years) via radiological detection (8), consistent with the age-related changes revealed in our current study. However, information regarding the direct relationship between SAD's radiological and spirometric manifestations in healthy adults is limited; hence, future research should focus on this topic.

This study found that in a specific healthy population in China, the age threshold for small airway function decline was 55 years. The intrinsic mechanisms may involve depletion of adult stem cell reservoirs, mitochondrial dysfunction, increased oxidative stress, and telomere shortening at this age (38). These changes can negatively affect neighboring cells, tissue structure, and extracellular matrix composition (38), causing dilation of the alveolar ducts and uniform enlargement of the alveolar air spaces (4). Another crucial element useful in stabilizing the alveoli is the secretion of surfactant by the cells lining the alveoli, which helps prevent alveolar collapse by reducing the surface tension within the alveoli. However, information on changes in surfactants with aging is limited. An animal study showed that surfactant levels remained unchanged with aging and exercise (39). In older individuals, alveolar enlargement results in reduced alveolar surface tension, causing a more compliant or distensible lung (4). In our study, all the aforementioned changes may have intensified by the age of 55 years. Pathological studies of the peritumoral tissue could provide supporting evidence from this viewpoint, and future research should focus on this area.

In our study, SAD was ruled out at over 55 years of age (NPV, 90.6%), a value that further increased to 93.5% after adjusting for covariates. Specifically, for screening SAD in a healthy Chinese population, age >55 years demonstrated a sensitivity, specificity, and positive predictive values of 89.2%, 53.8%, and 49.8%, respectively. These results suggest that using a 55-year age threshold leads to a lower misdiagnosis rate when differentiating SAD in healthy individuals. We also observed that healthy people aged 55 years or older exhibited lower FVC, FEV_1_, and FEV_1_/FVC values, although they were within the normal range (all *P* < 0.05), consistent with changes observed in asthma (22). This would be helpful in reducing the overdiagnosis of asthma or COPD in older patients aged 55 years or older, supporting a previous study showing that age-specific reference values should be considered for asthma or COPD diagnosis (22, 23, 40).

Considering the effects of aging on pulmonary function, we speculated that age-related lung changes, including dilated alveolar ducts and alveolar enlargement, may coexist with pathological changes in older adults with asthma or COPD. However, unlike patients with asthma or COPD, healthy older individuals do not have significant airway inflammation or changes in collagen and elastin content (4, 41). Elastic recoil loss in the lungs of healthy older individuals is a well-known change, similar to that observed in chronic asthma (42). Furthermore, older age is considered an important predictor of SAD in patients with asthma (37), which is associated with both a greater daily dose of inhaled corticosteroids (37) and a worse clinical expression of asthma (43). In asthmatics with FEV_1_ ≥ 80% predicted, the optimal cutoff values of small airway function variables for predicting a positive MCT were higher for patients aged below 55 years than for those aged 55 years or older (22). In patients with COPD, age-related SAD in healthy individuals may delay diagnosis. Therefore, strengthening early COPD detection initiatives through comprehensive screening programs is a critical priority. For older patients with COPD, more aggressive intervention measures might be needed to improve small airway function. Future research should focus on long-term follow-up studies to assess the changes in age-related SAD and how these changes influence COPD pathogenesis and progression. Early intervention measures for age-related SAD and their potential benefits in delaying COPD progression warrant further attention.

The statistical design of this study followed a progressive, step-by-step approach detailed in the methods. We demonstrated the reliability of our data calculations using an 80/20 split-sample cross-validation approach, where most indicators in the validation sample closely matched those in the entire model.

This study had several limitations. First, while smoking is an independent risk factor for SAD, we only included non-smokers to specifically clarify the effect of age on SAD. Consequently, our findings cannot be generalized to smokers alone; future studies should investigate the effect of age on SAD in smokers. Second, we only included 157 participants with SAD; therefore, studies with larger groups are needed to better represent the population. Third, given the different environmental conditions across various regions, the age at which the small airway function declines may also vary. Therefore, stratified analyses across different regions are necessary. Therefore, additional data are required for future studies. Fourth, this was a cross-sectional study; therefore, it cannot establish a causal relationship between SAD and age. Further longitudinal studies are required to verify a causal relationship between age and SAD status. Fifth, despite our efforts to control confounding factors by selecting a specific population, factors such as race, genetics, air quality, and physical activity may still affect the interpretation of our results. However, to the best of our knowledge, this is the first study in China to explore the age threshold for the physiological decline in small airway function. This provides valuable insights for the age-stratified diagnosis and treatment of asthma and COPD.

## Conclusions

Age emerged as a significant independent risk factor for SAD, with 55 years identified as a key threshold for screening SAD in healthy Chinese adults. This finding should aid in the age-stratified diagnosis and treatment of asthma and COPD. Future research should investigate age-related effects on SAD effects in smokers and diverse healthy populations across different regions of China to provide more comprehensive insights.

## Data Availability

The original contributions presented in the study are included in the article/Supplementary Material; further inquiries can be directed to the corresponding author.
